# Cigarette smoke induces β_2_-integrin-dependent neutrophil migration across human endothelium

**DOI:** 10.1186/1465-9921-12-75

**Published:** 2011-06-08

**Authors:** Saskia A Overbeek, Saskia Braber, Paul A J Henricks, Marije Kleinjan, Vera M Kamp, Niki A Georgiou, Johan Garssen, Aletta D Kraneveld, Gert Folkerts

**Affiliations:** 1 Division of Pharmacology, Utrecht Institute for Pharmaceutical Sciences, Faculty of Science, Utrecht University, Utrecht, The Netherlands; 2 Department of Respiratory Medicine, University Medical Center Utrecht, Utrecht, The Netherlands; 3 Danone Research - Centre for Specialised Nutrition, Wageningen, The Netherlands

## Abstract

**Background:**

Cigarette smoking induces peripheral inflammatory responses in all smokers and is the major risk factor for neutrophilic lung disease such as chronic obstructive pulmonary disease. The aim of this study was to investigate the effect of cigarette smoke on neutrophil migration and on β_2_-integrin activation and function in neutrophilic transmigration through endothelium.

**Methods and results:**

Utilizing freshly isolated human PMNs, the effect of cigarette smoke on migration and β_2_-integrin activation and function in neutrophilic transmigration was studied. In this report, we demonstrated that cigarette smoke extract (CSE) dose dependently induced migration of neutrophils *in vitro*. Moreover, CSE promoted neutrophil adherence to fibrinogen. Using functional blocking antibodies against CD11b and CD18, it was demonstrated that Mac-1 (CD11b/CD18) is responsible for the cigarette smoke-induced firm adhesion of neutrophils to fibrinogen. Furthermore, neutrophils transmigrated through endothelium by cigarette smoke due to the activation of β_2_-integrins, since pre-incubation of neutrophils with functional blocking antibodies against CD11b and CD18 attenuated this transmigration.

**Conclusion:**

This is the first study to describe that cigarette smoke extract induces a direct migratory effect on neutrophils and that CSE is an activator of β_2_-integrins on the cell surface. Blocking this activation of β_2_-integrins might be an important target in cigarette smoke induced neutrophilic diseases.

## Background

Neutrophils play a pivotal role in pulmonary inflammatory diseases, such as chronic obstructive pulmonary disease (COPD). COPD is a progressive disease, which is characterized by two major pathological processes, namely bronchitis and emphysema. The neutrophils accumulate in the affected tissues and contribute to the chronic inflammatory reaction, eventually leading to lung destruction [1,2]. It is generally accepted that cigarette smoking is the most important risk factor for the development of COPD. The WHO estimated that 73% of COPD mortality is related to smoking [3]. However, not only COPD patients have increased neutrophil counts in bronchoalveolar lavage fluid (BALF) and sputum [4-7]; increased neutrophil numbers are also observed in sputum of smokers without respiratory problems [5,8].

To migrate from the blood stream to the lung, neutrophils use a specific set of adhesion and chemokine receptors [9-11]. This multistep process of adhesive and migratory events includes selectin-mediated rolling, chemokine-induced activation of integrins and integrin-dependent firm adhesion leading to transendothelial migration. During rolling, neutrophils interact with the endothelial cell surface via selectins binding weakly to mucin-like structures bearing specific carbohydrate moieties [9-11]. These rolling interactions allow neutrophils to sense CXC-chemokines, such as CXCL1 and CXCL8, which are bound to the endothelial cells via heparin-like structures. These chemokines activate the neutrophils via G protein-coupled receptors, ultimately leading to firm adhesion. Neutrophil firm adhesion to endothelial cells is mediated via interaction between integrins, such as β_2_-integrins Lymphocyte Function-associated Antigen 1 (LFA-1; CD11a/CD18; α_L_β_2_) and Macrophage 1 antigen (Mac-1; CD11b/CD18; α_M_β_2_) on neutrophils and members of the immunoglobulin superfamily, such as ICAM-1 and ICAM-2 present on endothelial cells [9-12]. The β_2_-(CD18-)integrins are heterodimeric receptors, consisting of an α- and a β-chain that together form a ligand-binding head region with two legs that contain the transmembrane and cytoplasmic domains of each chain [13,14]. During inflammation, activation of these β_2_-integrins is essential, since it leads to a conformational change in structure, going from an inactive, low affinity state to an active, high-affinity state [13]. These conformational changes can be initiated via stimuli received by receptors for chemokines, cytokines or foreign antigens inducing intracellular signals (inside-out signaling) [14] and further strengthened by integrin clustering, transferring signals from the extracellular domain to the cytoplasm (outside-in signaling) [9].

The aim of this study was to investigate the effect of cigarette smoke on neutrophil movement and on β_2_-integrin activation and function in neutrophilic transmigration through endothelium. Our findings indicate that cigarette smoke has a direct effect on the migration of neutrophils and that cigarette smoke is an activator of β_2_-integrins on the cell surface, leading to firm adhesion and transmigration of neutrophils through endothelium.

## Methods

### Chemicals and reagents

2R4F reference cigarettes were from Kentucky Tobacco Research Institute (Lexington, KY, USA). Recombinant human CXCL8 was supplied by R&D Systems Europe Ltd. (Abingdon, United Kingdom). MgSO_4_, glucose and formyl Met-Leu-Phe (fMLP) was purchased from Sigma Aldrich Chemie BV (Zwijndrecht, the Netherlands). Human fibrinogen was supplied by Kordia Life Sciences (South Bend, IN, USA). Human Serum Albumin (HSA) was purchased from Sanquin Blood Bank (Amsterdam, The Netherlands). Trypsin-EDTA (0.05%), fibronectin from human plasma and calcein-AM (1 mM solution in DMSO) were obtained from Invitrogen (Breda, the Netherlands). TNF-α was from Endogen (Woburn, MA, USA). HEPES was obtained from Agros Organics (Geel, Belgium). NaCl, KCl, K_2_HPO_4_·3H_2_O, CaCl_2_, NH_4_Cl, KHCO_3_, EDTA (Triplex III) and trisodium citrate dihydrate were purchased from Merck KGaA (Darmstadt, Germany). Ficoll-Paque™ PLUS was purchased from GE Healthcare (Eindhoven, the Netherlands). PBS, Roswell Park Memorial Institute (RPMI) 1640 medium (without L-glutamine and phenol red) and Endothelial cell Growth Medium-2 (EGM-2) BulletKit were obtained from Lonza Verviers SPRL (Verviers, Belgium). FBS was from Perbio Science Nederland BV (Etten-Leur, the Netherlands).

### Antibodies

Functional blocking monoclonal antibodies IB4 (mouse IgG_2a_; α-CD18) and 44A (mouse IgG_1_; α-CD11b) were isolated from the supernatant of mouse hybridomas purchased from the American Type Culture Collection (ATCC). Mouse IgG_1 _and IgG_2a _isotype controls were obtained from R&D Systems Europe Ltd. (Abingdon, United Kingdom). Treatment of neutrophils with isotype control antibodies showed no difference in adhesion or transmigration as compared to buffer control, CSE control or fMLP control. Antibody concentrations and pre-incubation periods were adapted from [15-17].

### Cells

#### Isolation of human polymorphonuclear leukocytes

Human polymorphonuclear leukocytes (PMNs) were isolated as previously described [18] from fresh whole blood, for which healthy donors signed written consent forms. The study population consisted of 21 participants, 38.1% men and 61.9% women. The median age was 48 (range: 27-60). Resulting PMN preparations consisted of approximately 95-97% PMNs, based on PMNs physical parameters analyzed by flow cytometry and CD16 expression. The preparations were negative for CD14, meaning that the preparations did not contain monocytes.

#### HUVEC culture

Human umbilical vein endothelial cells (HUVECs) were isolated as previously described [19] and were provided by J.H. van Kats-Renaud (UMC Utrecht). The cells were cultured in EGM-2 and were grown to confluence in 5-7 days. Cell passages two to three were used for all experiments.

### Cigarette smoke extract (CSE)

CSE was prepared by using a smoking machine (Teague Enterprises, Davis, Ca, USA) as previously described [20]. Direct and side stream smoke from one 2R4F cigarette was directed via a tube through 5 ml PBS using a peristaltic pump. Subsequently, the optical density (OD) of this extract was determined using a spectrometer (UV-mini 1240, Shimadzu) measuring at wavelength 320 nm. Freshly prepared CSE was used in all experiments. Non-toxic solutions ranging from 0.03 to 0.24 OD were used in the present study as determined by Annexin-V staining and FACS analysis. These concentrations correspond to serum values of < 10 to 65 ng/ml cotinine, as measured with ELISA. These values of the breakdown product of nicotine are associated with light cigarette smoking or moderate passive exposure, according to the Foundation of Blood Research [21].

### Migration assay

The migration assay was performed as previously described [22,23]. Briefly, indicated reagents were placed in the bottom wells of a 3- μm 96-well polycarbonate filter plate (Millipore BV, Amsterdam, the Netherlands) in RPMI 1640 medium (without L-glutamine and phenol red) supplemented 1% heat-inactivated FBS. 2 × 10^5 ^PMNs, isolated from fresh whole blood, were added to the top portion. The plate was incubated for one hour at 37°C in 5% CO_2_. After removing the upper portion, the cells in each bottom well were counted for 30 seconds using a BD FACSCalibur Flow Cytometer with CellQuest Pro Software (version 5.2.1.).

### Adhesion assay

The adhesion assay was performed according to the procedure as described before [24,25] with some minor modifications. Briefly, wells of a 96-wells special optics black plate with a clear bottom (Sigma Aldrich Chemie BV, Zwijndrecht, the Netherlands), were coated with 100 μl fibrinogen (10 μg/ml) overnight at 4°C. Non-specific binding sites were blocked with blocking buffer (0.5% HSA in PBS) for 1 hour at 37°C. Freshly isolated PMNs were loaded with the non-fluorescent dye calcein-AM (1 μM) by incubating the cells for 30 minutes at 37°C. After labeling, the cells were washed with a HEPES incubation buffer (20 mM HEPES, 132 mM NaCl, 6 mM KCl, 1 mM MgSO_4_, 1.2 mM K_2_HPO_4 _supplemented with 5 mM glucose, 1 mM CaCl_2 _and 0.5% (w/v) HSA) and resuspended in this buffer to a concentration of 10^6 ^cells/ml. Subsequently, 5 × 10^4 ^cells were added to each well and incubated with indicated reagents for 30 minutes at 37°C. After incubation, fluorescence was measured at 485 nm excitation and 535 nm emission using a Mithras LB 940 fluorescence meter (Berthold Technologies, Belgium) before and after three washes with HEPES incubation buffer. The percentage adherence was determined by calculating the fluorescence after three washes as a percentage of the total fluorescence before washing.

### Transendothelial migration assay

The transendothelial migration of neutrophils was evaluated using 3 μm 24-transwell system filter plate (Sigma Aldrich Chemie BV, Zwijndrecht, the Netherlands). HUVECs were subcultured to confluent monolayers on the inserts precoated with fibronectin (0.5 μg/ml; 100 μl per insert) and pre-incubated with TNF-α (2 ng/ml; 100 μl per insert) for 5 hours. Freshly isolated PMNs were labelled with the non-fluorescent dye calcein-AM (3 μM) by incubating the cells for 20 minutes at room temperature under gentle agitation. Subsequently, 5 × 10^5 ^PMNs were added to the upper chamber whereas the indicated reagents were placed in the lower chambers. The plate was incubated for one hour at 37°C in 5% CO_2_. After removing the inserts, fluorescence was measured of 100 μl suspension of each lower chamber at 485 nm excitation and 535 nm emission using a Mithras LB 940 fluorometer (Berthold Technologies, Belgium). The percentage of transmigration was defined as the fluorescence value of the migrated PMNs divided by the value of total fluorescence, multiplied by 100.

### Animals

Male Balb/c mice, 5-6 weeks old were obtained from Charles River Laboratories and housed under controlled conditions in standard laboratory cages in the animal facility. They were provided free access to water and food. All *in vivo *experimental protocols were approved by the local Ethics Committee and were performed under strict governmental and international guidelines on animal experimentation.

### Cigarette smoke exposure

The one-week cigarette smoke exposure protocol was performed as previously described [26]. The mice were exposed in whole-body chambers to air (sham) or to air-diluted mainstream cigarette smoke from the reference cigarettes 2R4F using a peristaltic pump. Just before the experiments, filters were cut from the cigarettes. Each cigarette was smoked in 5 minutes at a rate of 5l/hour in a ratio with 60l/hour air. The mice were exposed to cigarette smoke using 5 cigarettes twice daily for five consecutive days, except for the first day when they were exposed to 3 cigarettes. The mice were sacrificed 16 hours after the last air or smoke exposure.

### Immunohistochemistry

Immunohistochemistry was performed according to the procedure as described before [26] with some minor modifications. Mice used for morphometric analysis, were sacrificed by an i.p. injection with an overdose of pentobarbital (Nembutal™, Ceva Santé Animale, Naaldwijk, The Netherlands). The left lung was dissected and fixed with 10% formalin for at least 24 hours, after which it was embedded in paraffin.

Paraffin sections were deparaffinized, endogenous peroxidase activity was blocked with 0.3% H_2_O_2 _(Merck, Darmstadt, Germany) in methanol for 30 minutes at room temperature and rehydrated in a graded ethanol series to PBS. For antigen retrieval, the slides were boiled in 10 mM citrate buffer (pH 6.0) for 10 minutes in a microwave. The slides were cooled down to room temperature, rinsed with PBS (3x) and blocked with 5% goat serum (Dakocytomation, Glostrup, Denmark) in 1% bovine serum albumin in PBS for 30 minutes at room temperature. Sections were incubated with the primary antibody (rabbit polyclonal CD11b antibody, ab75476, 1.1 μg/ml, Abcam) in 1% bovine serum albumin/PBS overnight at 4°C. The slides were rinsed with PBS (3x) and incubated with the biotinylated secondary antibody (goat-anti-rabbit, 1:200, Dakocytomation) in 1% bovine serum albumin/PBS for 45 minutes at room temperature. The slides were rinsed with PBS (3x) and the biotinylated proteins were visualized by incubation with streptavidin-biotin complex/horseradish peroxidase (Vectastain Elite ABC, Vector Laboratories) for 45 minutes at room temperature, followed by 0.015% H_2_O_2_/0.05% diaminobenzidene (Sigma, Schneldorf, Germany)/0.05 M Tris-HCl (pH 7.6) for 10 minutes at room temperature. Sections were counterstained with Mayers' haematoxylin (Merck), dehydrated and mounted in Permount (Fisher Scientific). Negative controls without the primary antibody and normal rabbit IgG (AB-105-C, R&D systems) were included as controls. Photomicrographs were taken with an Olympus BX50 microscope equipped with a Leica DFC 320 digital camera.

### Statistical analyses

For all statistical analyses, GraphPad Prism version 4.0 was used. Two-tailed paired Student *t*-tests were used for comparing two groups. For comparing three or more groups, the data were analyzed using a one-way repeated measures ANOVA followed by Tukey post hoc analysis. Data were considered significant at p < 0.05. All results are expressed as means ± SEM.

## Results

### CD11b expression in the airways of mice after 5 days smoke exposure

**Figure 1:**
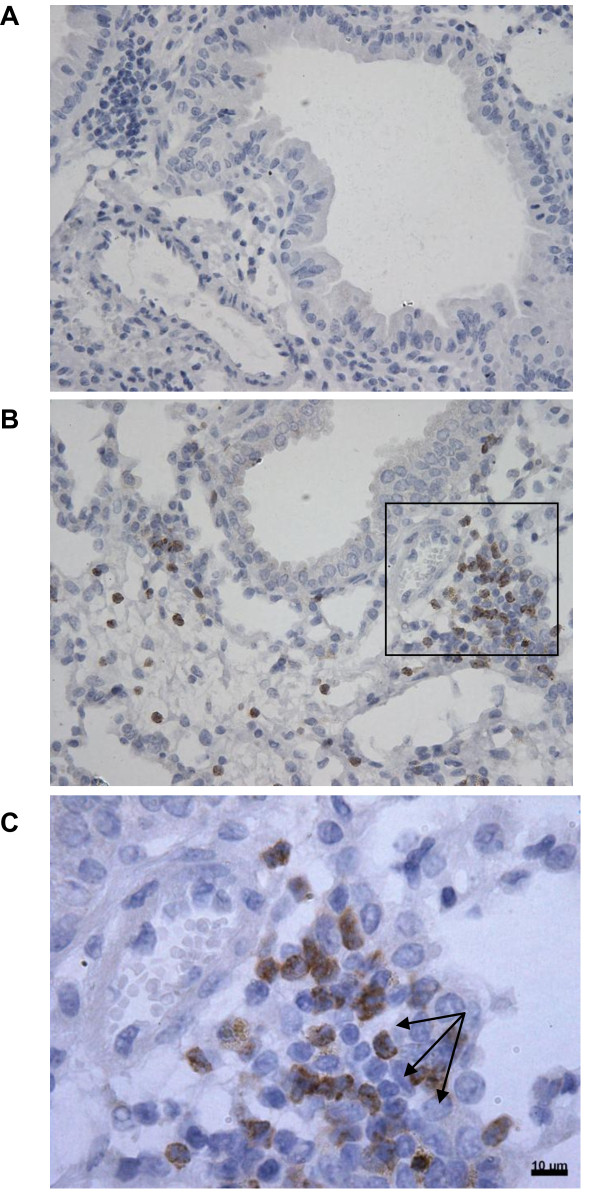
**CD11b expression in the lung after 1 week smoke exposure**. Representative photomicrographs (*n *= 3) of an immunohistological staining for CD11b (brown color, DAB staining) in lung tissue of air-exposed mice (A) and smoke-exposed mice (B and C (magnified from figure B)). CD11b expressing neutrophils (indicated with arrows in C) are present in mice exposed to cigarette smoke, whereas air exposed mice did not show an influx of these cells.

Cigarette smoke exposure to mice leads to a neutrophil influx in BALF and lung tissue [26]. To investigate the expression of CD11b *in vivo*, male Balb/C mice were exposed to air or smoke for 5 days and immunohistological staining for CD11b was performed on lung sections. Smoke exposure resulted in an increase of CD11b expressing cells (figure 1B), whereas air exposure did not result in CD11b positive cell accumulation in the lung (figure 1A). Focusing on the morphology of these CD11b expressing cells, it became clear that these cells were neutrophils since these cells showed segmented nuclei characteristic for neutrophils (figure 1C). These findings indicate that CD11b expressing neutrophils are present in the airways of mice exposed to cigarette smoke.

### Cigarette smoke extract induces migration of PMNs in vitro

**Figure 2:**
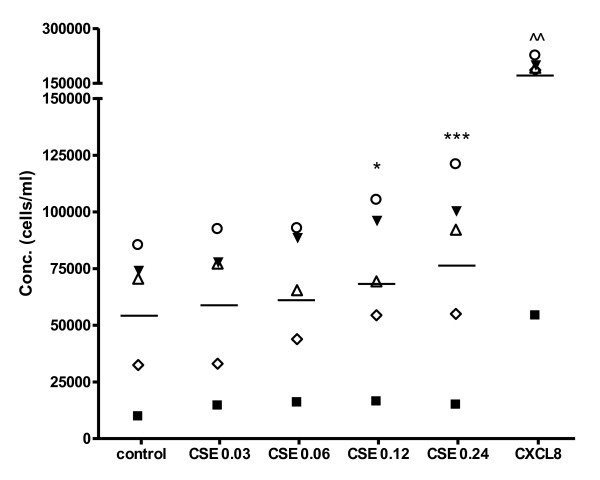
**Cigarette smoke extract induces migration of PMNs *in vitro***. 2 × 10^5 ^freshly isolated PMNs were placed in the top well and the cigarette smoke extract (CSE) was added to the bottom well of a 96-wells Millipore Filtration Plate System. After one hour incubation at 37°C in 5% CO_2_, the cells from each bottom well were counted. CSE (OD 0.03-0.24) induces migration of PMNs (*, p < 0.05; ***, p < 0.001 CSE vs. control). Legend: each symbol represents a different donor (N = 5, *n *= 3-9).

The in vivo findings described above, led us to the hypothesis that cigarette smoke may induce the migration of neutrophils. A transwell migration system was used to evaluate this effect of CSE on PMNs from fresh whole blood. After one hour incubation, the migration of PMNs to the lower chamber was quantified. For all experiments, non-toxic concentrations of CSE were used. CSE (OD 0.03-0.24) was dose-dependently chemotactic for PMNs (figure 2). CXCL8 (10 ng/ml) was tested as a positive control.

### Cigarette smoke extract induced adhesion of PMNs to fibrinogen is mediated by the activation of Mac-1

Neutrophils migrate from the blood into the extracellular matrix by activating β_2_-integrins, such as Mac-1 [9,10]. This led to the hypothesis that besides its migratory effect, cigarette smoke may activate the integrin Mac-1 on neutrophils, leading *in vivo *to a situation where more neutrophils transmigrate from the blood stream into the lung tissue.

**Figure 3:**
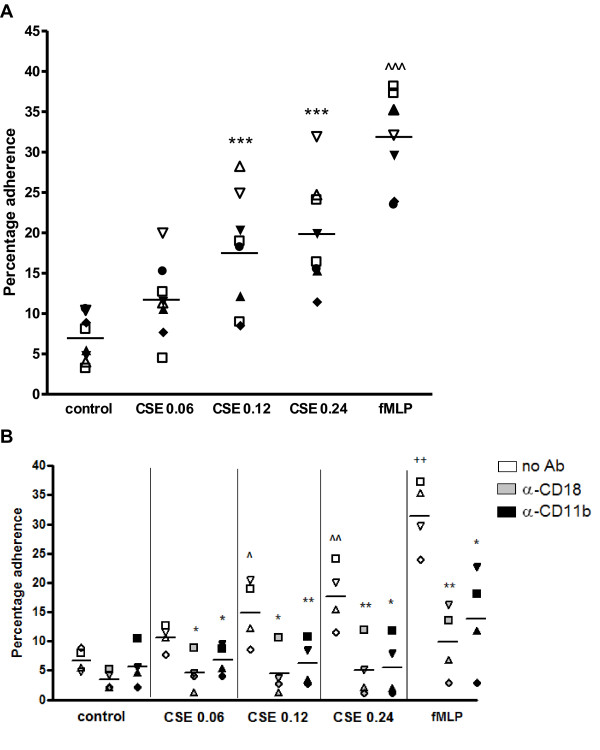
**CSE-induced adhesion of PMNs to fibrinogen is inhibited after pre-incubation with α-CD11b and α-CD18**. Calcein-AM labeled PMNs were added to fibrinogen-coated wells and incubated with indicated reagents for 30 minutes at 37°C. After incubation, fluorescence was measured before and after three washes with HEPES incubation buffer. (**A**) Cigarette smoke extract (CSE; OD 0.06-0.24) induces adhesion of PMNs to fibrinogen (***, p < 0.001 CSE vs. control. Legend: each symbol represents a different donor (N = 8, *n *= 3-8). (**B**) After labeling, PMNs were pre-incubated for 20 minutes with medium (white bars), α-CD18 (10 μg/ml; grey bars) or α-CD11b (10 μg/ml; black bars) at 37°C prior to incubation with medium or cigarette smoke extract (CSE) for 30 minutes at 37°C. Cigarette smoke extract (CSE; OD 0.06-0.24) induces adhesion of PMNs to fibrinogen (^, p < 0.05; ^^, p < 0.01 CSE vs. control), which is blocked by α-CD18 and α-CD11b (*, p < 0.05; **, p < 0.01 CSE α-CD11b/CD18 vs. CSE no Ab). FMLP (10^-8 ^M) induces adhesion of PMNs to fibrinogen (^++^, p < 0.01 fMLP vs. control), which is blocked by α-CD18 and α-CD11b (*, p < 0.05; **, p < 0.01 fMLP α-CD11b/CD18 vs. fMLP no Ab). Legend: each symbol represents a different donor (N = 4, *n *= 3).

To examine this hypothesis, the effect of CSE on the Mac-1-fibrinogen interaction was investigated. Incubating PMNs with CSE (OD = 0.06-0.24) caused a significant increase in adhesion to fibrinogen compared to medium incubation (figure 3A). Functionally blocking CD11b or CD18 on the PMN resulted in a complete inhibition of the CSE-induced activation and showed no significant difference with the control (figure 3B). FMLP (10^-8^M) was tested as a positive control.

### Functionally blocking CD11b and CD18 attenuates cigarette smoke extract induced transmigration of neutrophils through HUVECs

**Figure 4:**
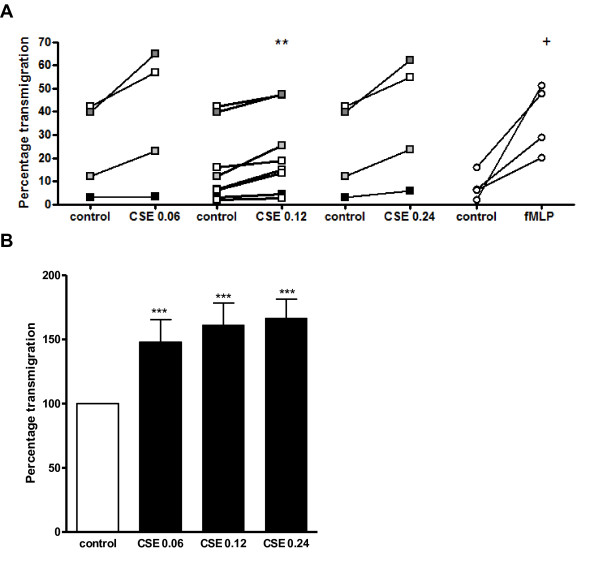
**CSE induces transmigration of neutrophils through HUVECs**. 5 × 10^5 ^calcein-AM labeled PMNs were placed in the top well on HUVEC layers and the indicated reagent (CSE or fMLP) was added to the bottom well of the transmigration system. After one hour incubation at 37°C in 5% CO_2_, fluorescence in 100 μl from each bottom well was measured. Data was standardized to the fluorescence of 100 μl PMNs (5 × 10^5 ^cells). (**A**) CSE (OD 0.06-0.24) induces transmigration of PMNs (**, p < 0.01 CSE vs. control). FMLP was tested as a positive control (^+^, p < 0.01 fMLP vs. control). Legend: each color represents a different donor (N = 4-8, *n *= 2). (**B**) Standardizing the data of figure 4A to an index where the transmigration under control situation is 100%, CSE incubation leads to approximately a 1.5-fold higher migratory capacity (***, p < 0.001 CSE vs. control).

Figures 2 and 3 show that cigarette smoke induces neutrophil movement and that it can activate β_2_-integrins on the PMN. A transmigration assay was performed to investigate the effect of cigarette smoke on the next step in neutrophil recruitment. Administration of the CSE did not affect the permeability of the HUVEC layer in this assay since the electrical resistance of the confluent HUVEC layer did not change after CSE exposure (Table 1). The transendothelial migration of PMNs was significantly higher in response to CSE (OD = 0.06-0.24) than to medium *in vitro *(figure 4A and 4B). Performing this assay in absence of the HUVEC layer resulted in a spontaneous transmigration of 38.97% ± 6.58%, whereas neutrophilic transmigration towards fMLP (10^-8 ^M) was 73.83% ± 4.63% (N = 4-6; data not shown). This leads to the conclusion that neutrophils actively migrate through an endothelial layer in response to CSE.

**Table 1:** Transendothelial electric resistance of the HUVEC layer

Antibody	**TER ( Ω × cm**^**2**^**) after medium exposure**	**TER ( Ω × cm**^**2**^**) after CSE exposure**
**No Ab**	158	148
**α-CD18**	155	149
**IgG**_**2a **_**isotype**	150	153
**α-CD11b**	153	157

No differences in transendothelial electrical resistance (TER) of the HUVEC layer were observed after antibody incubation or CSE (OD = 0.12) exposure as compared to medium exposure. The TER of filters alone was 82 Ω × cm^2^. Data in table 1 are representative of three separate experiments.

**Figure 5:**
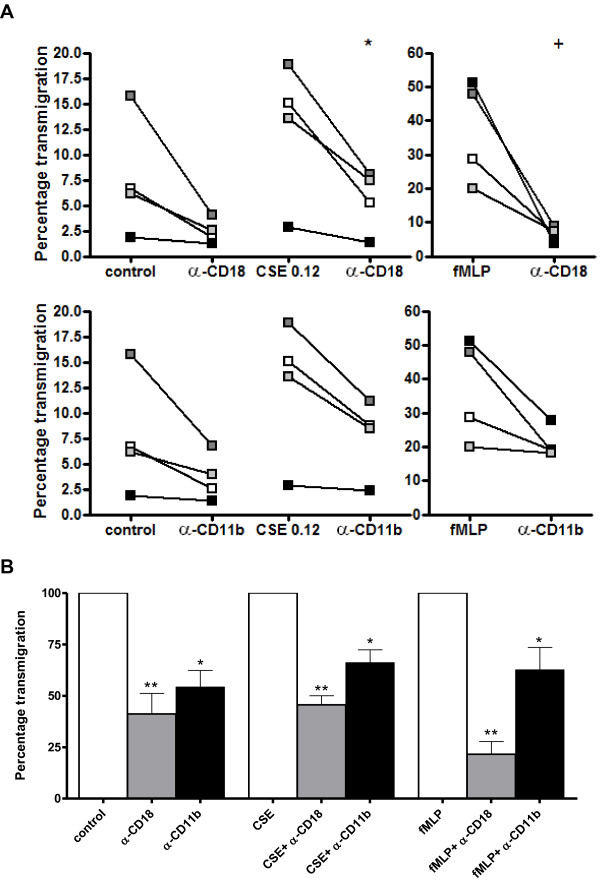
**CSE-induced transmigration of neutrophils through HUVECs is decreased after pre-incubation with α-CD11b or α-CD18**. (**A**) Prior to the transmigration assay, PMNs were pre-incubated for 20 minutes with medium, α-CD18 (10 μg/ml; upper panel) or α-CD11b (10 μg/ml; lower panel) at 37°C. CSE-induced transmigration of PMNs is decreased after pre-incubation with α-CD11b or α-CD18 (*, p < 0.05 CSE α-CD18 vs. CSE no Ab). FMLP (10^-8 ^M) was tested as a positive control (^+^, p < 0.01 fMLP vs. control). Legend: each color represents a different donor (N = 4, *n *= 2). (**B**) Standardizing the data of figure 5A to an index where the transmigration after CSE incubation is 100%, pre-incubation with α-CD11b or α-CD18 leads to a decrease in transmigration of approximately 35% and 50% resp. (*, p < 0.05; ** p < 0.01 CSE α-CD18/α-CD11b vs. CSE no Ab). Spontaneous transmigration of PMNs was also inhibited by blocking CD11b and CD18 (*, p < 0.05; ** p < 0.01 control α-CD18/α-CD11b vs. control no Ab). FMLP (10^-8 ^M) was tested as a positive control (*, p < 0.05; ** p < 0.01 fMLP α-CD18/α-CD11b vs. fMLP no Ab).

To assess the role of CD11b and CD18 in the transmigration of PMNs through HUVECs, PMNs were pre-incubated with antibodies against CD11b or CD18 after which the transmigration assay was performed. Functionally blocking CD11b or CD18 resulted in a decrease in CSE-induced transmigration of PMNs of approximately 35% and 50% resp. (figure 5A and 5B). FMLP (10^-8^M) was tested as a positive control.

## Discussion and conclusions

In this report, the direct effect of cigarette smoke on neutrophil migration and on β_2_-integrin activation and function in the endothelial transmigration of neutrophils was investigated. Exposing mice to cigarette smoke for 5 days resulted in the presence of CD11b expressing neutrophils in the lungs, showing that cigarette smoke attracts neutrophils. For a more in depth study, we exposed freshly isolated human neutrophils to CSE to study possible migratory effects and involvement of Mac-1. To our knowledge, this is the first study to show that CSE can induce the migration of neutrophils *in vitro*. Moreover, CSE activated the β_2_-integrin Mac-1 on the neutrophil leading to firm adhesion to fibrinogen. Furthermore, neutrophils transmigrate through endothelium in response to CSE via the activation of β_2_-integrins, since functionally blocking CD11b and CD18 decreased this transmigration. Taken together, our data provide evidence for a critical role of β_2_-integrins in the firm adhesion and transmigration of neutrophils in response to cigarette smoke *in vitro *and *in vivo*.

Cigarette smoke consists of more than 4000 compounds, known to be mutagenic, carcinogenic, antigenic and cytotoxic [27,28] and it induces a peripheral inflammatory response in all smokers [29]. This is reflected in increased macrophage and neutrophil numbers, changes in expression surface markers, elevated levels of chemokines (amongst which are CXCL1 and CXCL8) and cytokines (such as TNF-α and IL-1β) and increased production of proteolytic enzymes by different immune cells, such as macrophages and neutrophils [18,28,30].

Exposing mice for five days to cigarette smoke results in an influx of CD11b expressing cells. We concluded that these cells were neutrophils due to the characteristic segmented nuclei. Other studies also observed increased numbers of neutrophils in BALF and lung tissue after one week [26,31] and five months cigarette smoke exposure in mice [32]. These *in vivo *findings led to the hypothesis that cigarette smoke may has as a direct migratory effect. Interestingly, it has recently been suggested by Barnes that smoke may induce neutrophil movement [33]. In the present study, we confirm this hypothesis and demonstrate that CSE induces the migration of neutrophils. Although it has been reported that nicotine, a major constituent of cigarette smoke, can have a chemotactic effect on neutrophils [34], other groups found no effect or even an inhibitory effect [35-37] or describe this effect as very weak and state that nicotine enhances neutrophil responsiveness to other chemo-attractants [38]. Another major constituent of smoke, acrolein, inhibits chemotaxis [36,39]. Since CSE consist of more than 4000 components, it is likely that other chemical compounds or combinations of soluble compounds are responsible for the chemotactic effect on neutrophils.

To our knowledge, we are the first to show a direct migratory effect of CSE. Other groups have investigated the priming effect of cigarette smoke. Bridges and Hsieh demonstrated that water-soluble fractions (WSF) and cigarette smoke condensates (CSC) were able to inhibit endotoxin-activated serum-induced chemotaxis of neutrophils [40]. Selby et al. described that exposure of human neutrophils *in vitro *to cigarette smoke resulted in impaired cell spreading and chemokinesis, but did not influence zymosan-activated serum-induced chemotaxis [41]. However, both groups did not investigate the direct migratory effect of cigarette smoke on neutrophils. The inhibitory effects of cigarette smoke on activated serum-induced chemotaxis of neutrophils, as described in the studies of Bridges and Hsieh and Selby et al. [40,41], may be explained by the fact that adding the cigarette smoke to the neutrophils leads to immobilization of the cells; the cells are attracted by the cigarette smoke in the upper chamber and do not actively need to go to the lower chamber.

Neutrophils use β_2_-integrins, such as Mac-1, for nearly every step in transmigration [13]. During inflammation, β_2_-integrin on the neutrophil are activated by chemokines such as CXCL8, leading to neutrophilic firm adhesion to endothelial cells and subsequently to transmigration. This activation is essential, since it leads to a conformational change in structure, going from an inactive, low affinity state to an active, high-affinity state [13]. We show that CSE activates β_2_-integrins on the neutrophil leading to firm adhesion to fibrinogen and transmigration through an endothelial cell layer, since functionally blocking CD11b and CD18 decreased these effects of cigarette smoke. Consistent with previous studies describing β_2_-integrin expression patterns of smokers [29,42,43], we found that CSE-induced adhesion and transmigration of neutrophils are the result of β_2_-integrin activation, and more specifically Mac-1 activation. It may be possible that cigarette smoke exposure leads to an up-regulation of β_2_-integrin expression on the neutrophil. Koethe et al. described that pre-incubating PMNs with CSC resulted in a 2.5 fold increase in CD11b/CD18 expression as compared to the control [44]. However, Selby et al. reported that acute *in vivo *cigarette smoking of up to 4 cigarettes did not change the expression patterns of CD18 on the neutrophils [41]. From these results it can be suggested that besides the activation of β_2_-integrins, CSE incubation may result in an up-regulation of the CD11b/CD18 expression.

Cigarette smoke enhances the expression of E- and P-selectin [45] and ICAM-1 and VCAM-1 [46] on HUVECs, thereby increasing rolling interactions and subsequent firm adhesion of neutrophils to HUVECs via β_2_-integrins Mac-1 and LFA-1 and β_1_-integrin VLA-4 [9]. In our experiments, neutrophils migrated through an endothelial cell layer in response to CSE. Administration of cigarette smoke did not affect the permeability of the HUVEC layer in the transmigration assay since the electrical resistance of the confluent HUVEC layer did not change before and after CSE exposure (Table 1). This leads to the conclusion that neutrophils actively migrate through the layer in response to cigarette smoke.

Although CXCL8 has a stronger migratory effect and fMLP is a stronger activator of β_2_-integrins, cigarette smoke exposure leads to a 1.5 fold increase in migration and to a 1.5 to 3 fold increase in adhesion as compared to control. Mortaz and colleagues have recently shown that CSE exposure to human neutrophils results in the release of CXCL8 [18]. Furthermore, earlier studies have demonstrated that exposure of neutrophils to CXCL8 leads to the phosphorylation of focal adhesion kinase (FAK) and CXCL8 induces FAK cellular redistribution [47]. FAK activation is involved in cellular adhesion and spreading processes of neutrophils [48]. Thus in the *in vivo *situation, we cannot rule out the additional chemotactic effects of cigarette smoke-induced released inflammatory mediators from for example epithelial cells, pulmonary neutrophils and resident macrophages.

Taken together, this is the first study that describes CSE as a potent inducer of neutrophil movement. Besides this migratory effect, CSE plays a critical role in β_2_-integrin activation, which leads to an increase in firm adhesion and transmigration through HUVECs. These observations may contribute to a better understanding of neutrophilic transmigration in smokers and COPD patients and potentially offer a new target in disease management.

## List of Abbreviations

ANOVA: analysis of variance; BALF: bronchoalveolar lavage fluid; COPD: chronic obstructive pulmonary disease; CSE: cigarette smoke extract; DMSO: dimethyl sulfoxide;EGM-2: endothelial cell growth medium-2; FBS: fetal bovine serum; fMLP: formyl-Methionyl-Leucyl-Phenylalanine; HSA: human serum albumin; HUVECs: human umbilical vein endothelial cells; ICAM-1/2: inter-cellular adhesion molecule-1/2; LFA-1: lymphocyte function-associated antigen 1; CD11a/CD18; α_L_β_2_; Mac-1: macrophage 1 antigen; CD11b/CD18; α_M_β_2_; OD: optical density; PBS: phosphate buffered saline; PMN: polymorphonuclear leukocyte; RPMI: Roswell Park Memorial Institute; SEM: standard error of the mean; TNF-α: tumor necrosis factor-alpha.

## Competing interests

The authors declare that they have no competing interests.

## Authors' contributions

SAO conducted most of the study and was involved in the design of the study, the analysis and interpretation of the data and the writing of the manuscript. SB conducted part of the study and reviewed the manuscript. MK and VMK assisted in the study. NAG and JG participated in the design of the study and reviewed the manuscript. PAJH, ADK and GF obtained the research support and participated in the design of the study, the interpretation of the data and reviewing of the manuscript. All authors read and approved the final manuscript.
